# Research on Plasma Characteristics of High-Power Impulse Magnetron Sputtering Ti-Nb-Cr Target and Its Effect on Film Properties

**DOI:** 10.3390/ma19091710

**Published:** 2026-04-23

**Authors:** Changzi Chen, Yantao Li, Donglin Ma, Quanxin Jiang, Jingjing Peng, Jianfei Wang

**Affiliations:** 1 College of Intelligent Manufacturing, Jingchu University of Technology, Jingmen 448000, China; ccz198311@163.com (C.C.); jqx@jcut.edu.cn (Q.J.); jingjinggl@163.com (J.P.); 2 School of Intelligent Manufacturing, Mianyang Teachers’ College, Mianyang 621016, China; liyantao1992@126.com; 3 College of Physics and Electronic Engineering, Chengdu Normal University, Chengdu 611130, China; mdl208115@163.com

**Keywords:** high-power impulse magnetron sputtering, TiNbCr film, ion atomic arrival ratio, mechanical properties, corrosion resistance

## Abstract

High-power impulse magnetron sputtering (HiPIMS) technology was used to deposit Ti-Nb-Cr films on Si (100) and 316L substrates by changing the peak power of the Ti-Nb-Cr target. Optical emission spectroscopy (OES) was used to study the effect of peak power on the ion atomic arrival ratio in front of the substrate. Experimental instruments such as an X-ray diffraction (XRD) device, scanning electron microscope (SEM), transmission electron microscope (TEM), nanohardness tester, ball-disk reciprocating friction machine, and electrochemical workstation were used to study the effects of the atomic arrival ratio of Ti, Nb, and Cr ions on the microstructure, mechanical properties, and corrosion resistance of Ti-Nb-Cr films. The results show that when the peak power is 67.84 kW, the ion atomic arrival ratio of Ti reaches 47.57%, the ion atomic arrival ratio of Nb reaches 39.41%, and the ion atomic arrival ratio of Cr reaches 10.6%. The ion atomic arrival ratio is doubled compared to the peak power of 51.04 kW. The films prepared at different peak powers all show diffraction peaks of the BCC structure. At high power levels, the TiNbCr films exhibit reduced residual compressive stress, although this may be accompanied by lower hardness and wear resistance.

## 1. Introduction

In recent years, multicomponent alloys based on refractory elements such as Ti, Nb, Cr, and Zr have attracted considerable research interest owing to their exceptional mechanical properties, wear and corrosion resistance, and thermal stability at elevated temperatures [1,2,3]. Machine learning approaches have emerged as powerful tools for the computational design of such alloys, enabling the prediction of optimal elemental combinations for targeted performance [4]. For instance, Ta_57_Ti_17_Zr_15_Si_11_ alloys with specific compositional ratios exhibit promising biocompatibility for orthopedic implant applications [5], while Cu-Ti-Nb coatings demonstrate superior hardness and tribological performance [6]. These ternary and quaternary systems (such as TaNbTiW), often classified as medium-entropy alloys (MEAs), typically crystallize in single-phase BCC, FCC, or dual-phase BCC/FCC configurations [4].

Titanium exhibits intrinsic advantages, including exceptional biocompatibility and a high melting point. Following the “cocktail effect” principle [7,8], chromium addition enhances corrosion resistance, whereas niobium improves thermal stability at elevated temperatures. The synergistic combination of these elements yields Ti-Nb-Cr medium-entropy alloys with superior integrated performance. Conventional preparation methods for multi-component alloy films include thermal spraying, arc ion plating, and direct current magnetron sputtering [9,10]. More recently, high-power impulse magnetron sputtering (HiPIMS) has gained widespread adoption for multi-alloy film deposition, attributed to its distinctive capabilities of delivering high peak-power densities and generating highly ionized plasma fluxes [11,12,13,14,15,16,17].

In this study, Ti-Nb-Cr films were deposited using HiPIMS to investigate how the ionization degree of sputtered species influences microstructural development and functional properties. Specifically, the ion-to-atom arrival ratio was modulated by varying the peak power through discrete trigger-voltage settings (700, 800, and 900 V), enabling systematic evaluation of its effects on mechanical integrity and corrosion resistance.

## 2. Materials and Methods

### 2.1. Thin-Film Deposition

Ti-Nb-Cr thin films were deposited on Si (100) wafers and 316L stainless steel substrates using an unbalanced magnetron sputtering system [18]. The cylindrical vacuum chamber, 500 mm in diameter and height, was equipped with a custom-fabricated three-element target assembly. This target comprised high-purity Ti (≥99.9%), Nb (≥99.9%), and Cr (≥99.9%) segments arranged in a periodic arrangement pattern with a mass ratio of approximately 4:3:1.5. The target surface dimensions were 150 × 125 mm^2^.

Prior to deposition, Si and stainless steel substrates were ultrasonically cleaned in acetone and ethanol sequentially before mounting on a rotatable substrate holder within the vacuum chamber. The chamber was evacuated to a base pressure of 1.0 × 10^−3^ Pa, after which high-purity argon (99.999%) was introduced as the sputtering gas. The target-to-substrate distance was fixed at 60 mm. To clean the target, a metal plate was used to mask the substrate during the cleaning process to avoid unwanted deposition, and the target was sputtered with Ar^+^ ions for 5 min. Subsequently, the target was masked while the substrate was sputtered for 10 min. Film growth was initiated using a HiPIMS power supply (HPS-450D, Chengdu Pulsetech Electrical Co., Chengdu, China), with detailed processing parameters summarized in Table 1. Discharge characteristics were diagnosed in situ by monitoring target voltage and current waveforms using a high-voltage probe (Tektronix P5100, Tektronix, Inc., Beaverton, OR, USA) and a wide-band current transformer (Pearson 411, Pearson Electronics, Inc., Palo Alto, CA, USA), with signals acquired by a digital storage oscilloscope (Tektronix TDS220, Tektronix, Inc., Beaverton, OR, USA) [19].

### 2.2. Thin-Film Characterization and Testing

Film thickness and residual stress were quantified using a surface profilometer (Ambios XP-2, Ambios Technology, Inc., Santa Cruz, CA, USA). The phase constitution and microstructure of Ti-Nb-Cr films deposited on Si substrates were characterized by X-ray diffraction (XRD, Philips X’Pert, Eindhoven, The Netherlands). XRD measurements were performed in θ–2θ geometry using Cu Kα radiation generated at 40 kV and 40 mA and then characterized by transmission electron microscopy (TEM, FEI Tecnai, Hillsboro, OR, USA). Chemical composition was determined by energy-dispersive X-ray spectroscopy (EDS, Oxford Instruments, Abingdon, UK). Surface and cross-sectional morphologies were examined using a field-emission scanning electron microscope (FE-SEM, Zeiss Sigma 300, Oberkochen, Germany). Mechanical properties, specifically hardness and elastic modulus, were assessed by instrumented nanoindentation (Anton Paar CSM UNHT, Baden, Switzerland) with a maximum applied load of 20 mN and a loading/unloading rate of 40 mN/min.

Tribological performance was evaluated using a ball-on-disc reciprocating tribometer (CSEM, Neuchâtel, Switzerland) under controlled environmental conditions (55% relative humidity, 12 ± 1 °C). A GCr15 bearing steel ball (6 mm in diameter) served as the counterbody, with tests conducted at an applied load of 0.5 N and a linear sliding speed of 3.77 cm/s over a total sliding distance of 40 m (1000 reciprocating cycles). For comparative assessment, an uncoated 316L stainless steel substrate was subjected to identical tribological testing. Wear track profiles and worn surface morphologies were quantified using stylus profilometry (Ambios XP-2, Ambios Technology, Inc., Santa Cruz, CA, USA) and optical microscopy (SDPTOP MX6R, Ningbo Sunny Instruments Co., Ltd., Ningbo, China) [20].

## 3. Results and Discussion

### 3.1. Target Discharge and Plasma Characteristics

**Figure 1:**
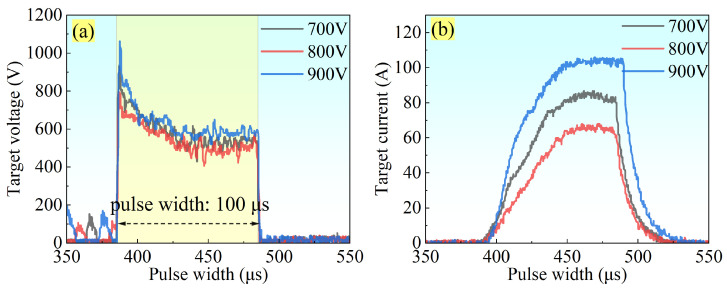
(**a**) Discharge voltage and (**b**) discharge current of the Ti-Nb-Cr target at different peak powers.

The temporal evolution of discharge voltage and current for the Ti-Nb-Cr target under varying peak-power conditions is presented in Figure 1. As shown in Figure 1a,b, peak currents of 88, 68, and 106 A were recorded at peak powers of 51.04, 39.44, and 67.84 kW, respectively. Notably, the peak current exhibited a monotonic increase with applied voltage, reaching its maximum at 900 V (67.84 kW). This trend can be attributed to enhanced Ar^+^ ion bombardment of the target surface at elevated voltages, which intensifies electron-neutral collisions in the near-target region, thereby generating substantial secondary electron and ion fluxes [21]. The resultant superposition of ion and secondary electron currents contributes to the observed increase in discharge current. Furthermore, Figure 1b reveals that the steepest current rise occurs at 67.84 kW (900 V). This pronounced rising-edge slope reflects higher secondary electron production and consequently elevated electron densities in the ionization zone, as the current waveform’s rising rate scales positively with electron density [22].

**Figure 2:**
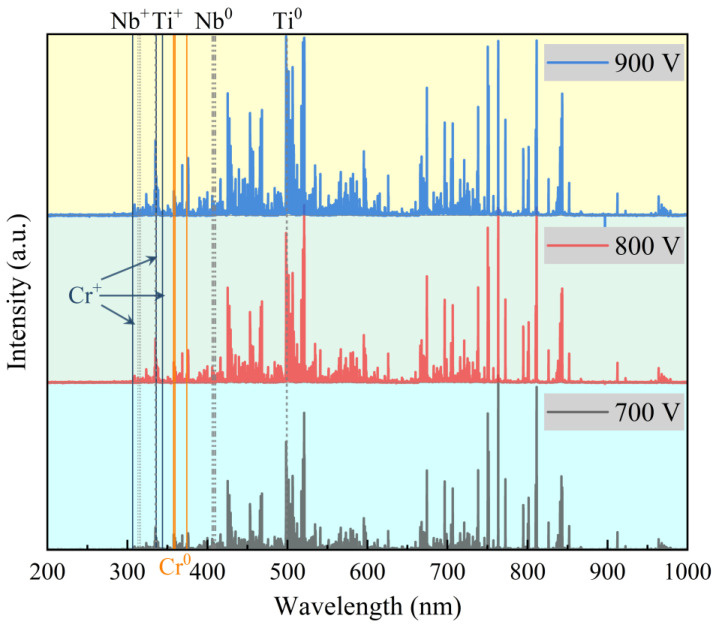
Emission spectral intensity values of plasma components at different trigger voltage rates.

Optical emission spectra acquired 10 mm from the substrate surface under varying peak power conditions are presented in Figure 2. The plasma emission was dominated by characteristic lines of ionized and neutral species, specifically Ti^+^ (334.83, 334.89, and 336.09 nm), Ti^0^ (498.2, 499.13, and 499.97 nm), Nb^+^ (313.06, 314.55, and 316.33 nm), Nb^0^ (405.9, 407.94, and 410.08 nm), Cr^+^ (306.55, 335.85, and 343.79 nm), Cr^0^ (357.85, 359.34, and 374.1 nm), Ar^+^, and Ar^0^. Eighteen principal spectral lines were selected from these characteristic peaks to quantify plasma composition, and ion-to-atom ratios were subsequently derived from the integrated line intensities following established spectroscopic methods [23,24].

**Figure 3:**
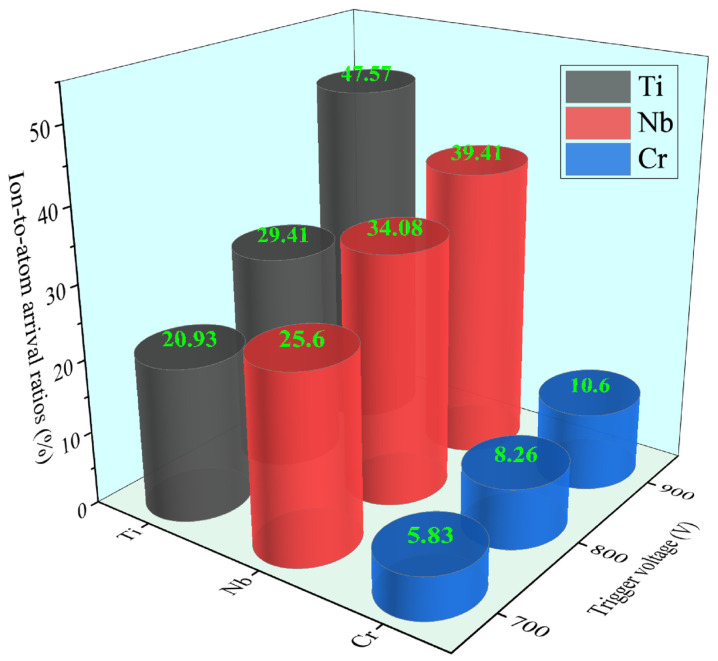
Ion-to-atom arrival ratios for Ti, Nb, and Cr as functions of different trigger voltage rates.

The ion-to-atom arrival ratios for Ti, Nb, and Cr as functions of trigger voltage are summarized in Figure 3. At trigger voltage rates of 700 V, 800 V, and 900 V, the Ti ionization degrees were 20.93%, 29.41%, and 47.57%, respectively, while those for Nb reached 25.6%, 34.08%, and 39.41%, and Cr attained 5.83%, 8.26%, and 10.6%.

Notably, all three elements exhibited their maximum ionization ratios at the highest peak power (67.84 kW). Plasma ionization is governed primarily by electron-impact ionization and Penning ionization [25]. Electron collisions with metal atoms, together with interactions between metastable Ar^+^ species and metal neutrals, generate substantial Ti^+^, Nb^+^, and Cr^+^ fluxes. Elevated peak power enhances secondary electron emission near the target surface, thereby intensifying collisional ionization and increasing ion-to-atom ratios. Furthermore, according to the self-sputtering mechanism in HiPIMS discharges [26,27], the increasing metal ion fraction progressively displaces Ar^+^ as the dominant sputtering species, with a significant portion of metal ions returning to the target to sustain self-sputtering—an effect that further amplifies the ionization degree.

### 3.2. Microstructure and Composition of Ti-Nb-Cr Thin Films

**Figure 4:**
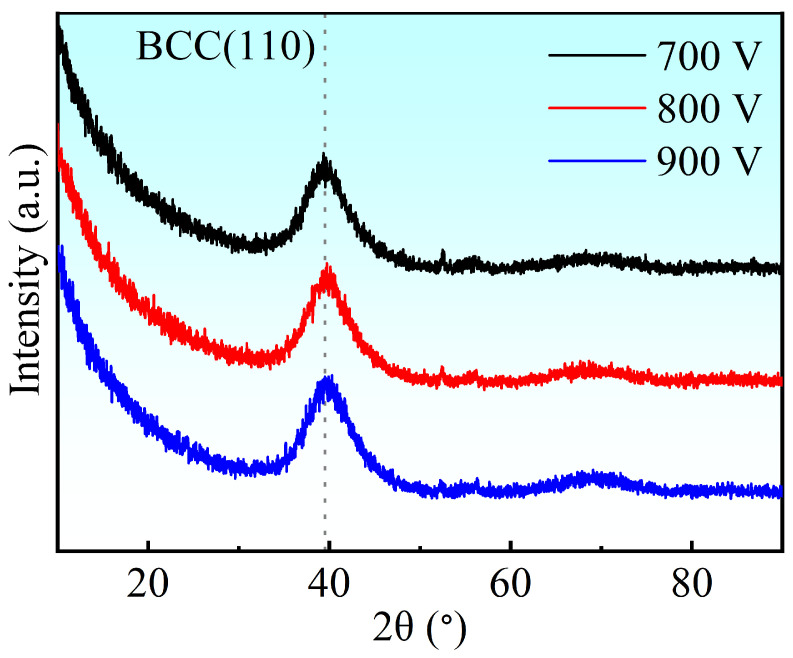
XRD patterns of TiNbCr films with different trigger voltages.

**Figure 5:**
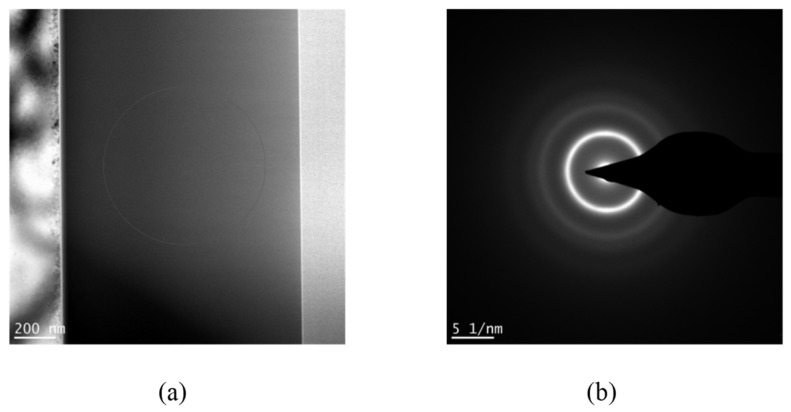
Cross-sectional TEM analysis of Ti-Nb-Cr film deposited by HiPIMS at 900 V: (**a**) bright-field image showing the section morphology and (**b**) selected-area electron diffraction (SAED) pattern exhibiting concentric diffuse halos indicative of an amorphous structure.

The phase evolution of Ti-Nb-Cr films as a function of trigger voltage is presented in Figure 4. The broad diffraction peak centered at ~39° corresponds to the (110) plane of the BCC solid solution phase [28], consistent with β-Ti (JCPDS 44-1288), Nb (JCPDS 35-0789), and Cr (JCPDS 06-0694). The absence of sharp Bragg peaks suggests an amorphous or nanocrystalline microstructure. Cross-sectional TEM analysis of the film deposited at 900 V reveals a featureless morphology (Figure 5a), while the corresponding selected-area electron diffraction (SAED) pattern (Figure 5b) displays concentric diffuse halos characteristic of an amorphous-dominant structure containing embedded nanocrystalline features.

**Figure 6:**
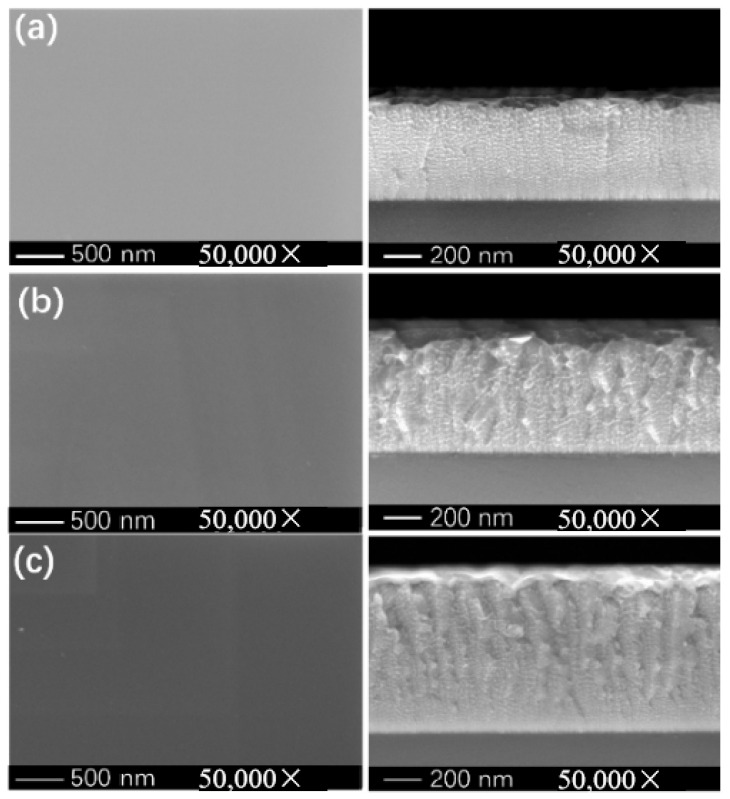
Section morphology of Ti-Nb-Cr films deposited at different trigger voltages: (**a**) 700 V, (**b**) 800 V, and (**c**) 900 V.

**Figure 7:**
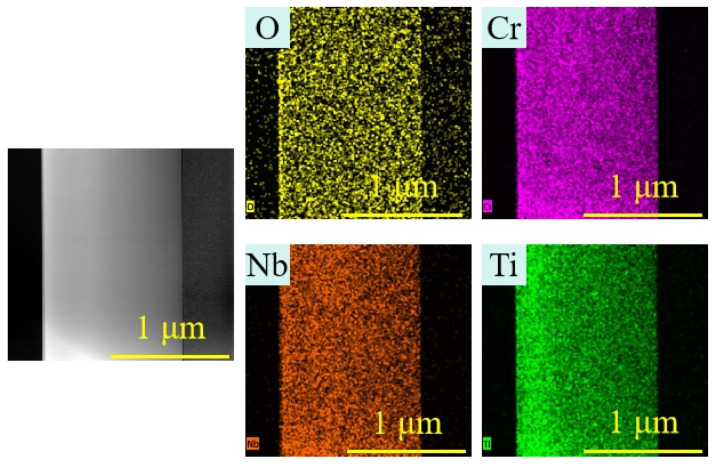
TEM of a section of TiNbCr film deposited by HiPIMS at 67.84 kW; composition distribution at the center (mapping).

Surface and cross-sectional morphologies of Ti-Nb-Cr films deposited at varying peak powers are presented in Figure 6. All films exhibit dense, featureless surface topographies without apparent porosity or defects. There are columnar structures in Figure 6b,c. Energy-dispersive X-ray spectroscopy (EDS) mapping of the film center region (Figure 7) indicates minor oxygen incorporation, with Ti, Nb, and Cr exhibiting dense, homogeneous spatial distributions throughout the film thickness.

### 3.3. Mechanical Properties of Ti-Nb-Cr Thin Film

**Figure 8:**
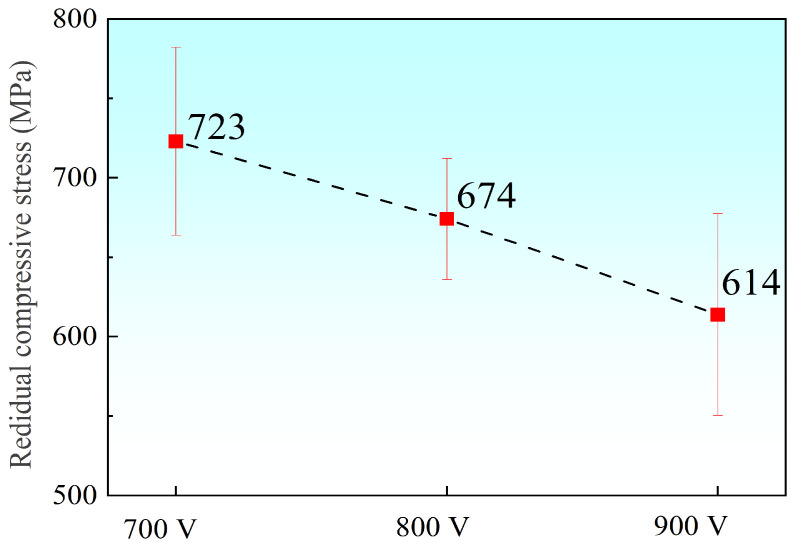
Residual compressive stress of Ti-Nb-Cr films with different trigger voltages.

Figure 8 illustrates the evolution of residual compressive stress in the Ti-Nb-Cr films as a function of trigger voltage. The stress exhibits a monotonic decrease, dropping from 723 MPa to 614 MPa with increasing power. This reduction is attributed to the enhanced ionization rate at higher peak powers, which intensifies the bombardment of the substrate by high-energy particles. Consequently, the substrate surface temperature rises, promoting increased atomic mobility. This thermal activation facilitates structural relaxation, effectively mitigating the intrinsic residual compressive stress within the film.

**Figure 9:**
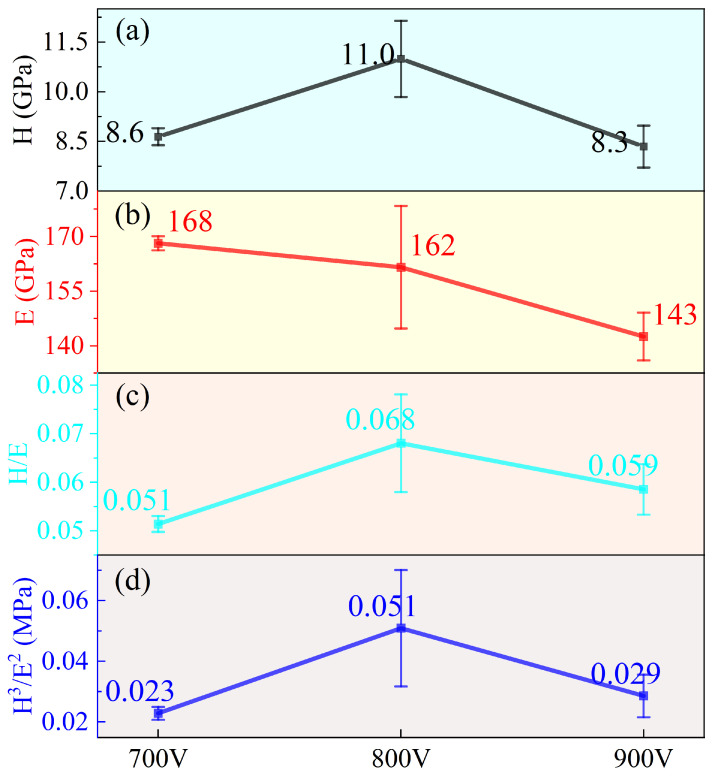
(**a**) Hardness H, (**b**) modulus E, (**c**) *H*/*E* and (**d**) *H*^3^/*E*^2^ values of TiNbCr films with different trigger voltages.

The ratios *H*/*E* and *H*^3^/*E*^2^ are widely employed as indicators of thin film toughness [29,30]. Specifically, *H*/*E* correlates with elastic strain recovery, while *H*^3^/*E*^2^ reflects resistance to plastic deformation. Figure 9 presents the hardness (H), elastic modulus (E), and the derived *H*/*E* and *H*^3^/*E*^2^ ratios for Ti-Nb-Cr films deposited at various trigger voltages. The hardness exhibits a nonmonotonic trend: it rises from 8.6 GPa at 700 V to a peak of 11.0 GPa at 800 V before declining to 8.3 GPa at 900 V. This reduction at higher voltage is likely associated with the relaxation of residual compressive stress discussed previously. In contrast, the elastic modulus demonstrates a gradual decrease with increasing ionization degree. The elastic modulus mainly depends on atomic bonding, phase structure, densification, and residual stress, rather than changing synchronously with hardness. At 800 V, the moderate ion flux induces structural relaxation and atomic rearrangement, which slightly reduces the elastic modulus but significantly improves hardness, leading to the optimal *H*/*E* and *H*^3^/*E*^2^ ratios. Thus, 800 V delivers the best comprehensive mechanical properties, even though the elastic modulus is not the maximum.

As derived from Figure 9, the *H*/*E* ratios for the Ti-Nb-Cr films deposited at different trigger voltages are 0.051, 0.068, and 0.058, respectively. The initial increase in the *H*/*E* ratio (from 0.051 to 0.068) suggests an enhancement in elastic strain recovery and overall film toughness as the ionization rate rises. Conversely, the subsequent decline at higher voltage rates indicates a reduction in these properties. Similarly, the *H*^3^/*E*^2^ ratios follow a trend of 0.023, 0.051, and 0.029. The peak value of 0.051 at the intermediate voltage signifies an optimal resistance to plastic deformation, while the decrease at 900 V implies a weakening of this resistance, consistent with the observed drop in hardness.

### 3.4. Tribology Properties of Ti-Nb-Cr Thin Films

**Figure 10:**
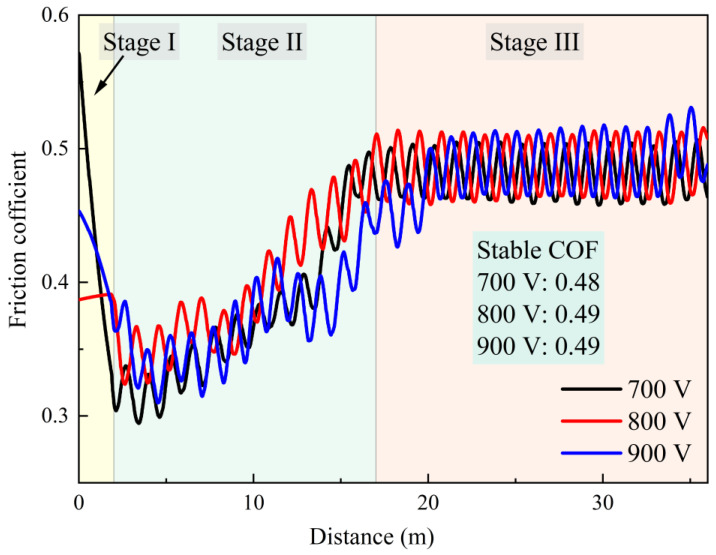
Friction coefficient of Ti-Nb-Cr films with different trigger voltages.

Figure 10 illustrates the friction coefficients of Ti-Nb-Cr films deposited at various trigger voltages. Notably, all three samples exhibited three distinct wear stages. Stage I (running-in stage) was characterized by a rapid removal of microscopic asperities on the contacting surfaces, resulting in an initially high friction coefficient that quickly declined. Subsequently, the process entered Stage II, where the progressive wear of the upper-ball specimen led to a widening of the wear track and an expansion of the macroscopic contact area, causing a gradual increase in the friction coefficient. Finally, the system reached Stage III (steady-state wear), during which the wear track width remained approximately constant while the depth continued to increase, stabilizing the friction coefficient. It is also evident from the figure that the steady-state friction coefficients for all three samples converged within a narrow range of 0.48 to 0.49. This consistency indicates that the interfacial friction and wear mechanism remain stable under different trigger voltages. Since the steady-state friction coefficient is directly governed by the composition, particle size, and distribution of wear debris within the contact zone, it can be reasonably inferred that the composition and particle size of the wear debris generated under these different voltage conditions are fundamentally similar [31,32].

**Figure 11:**
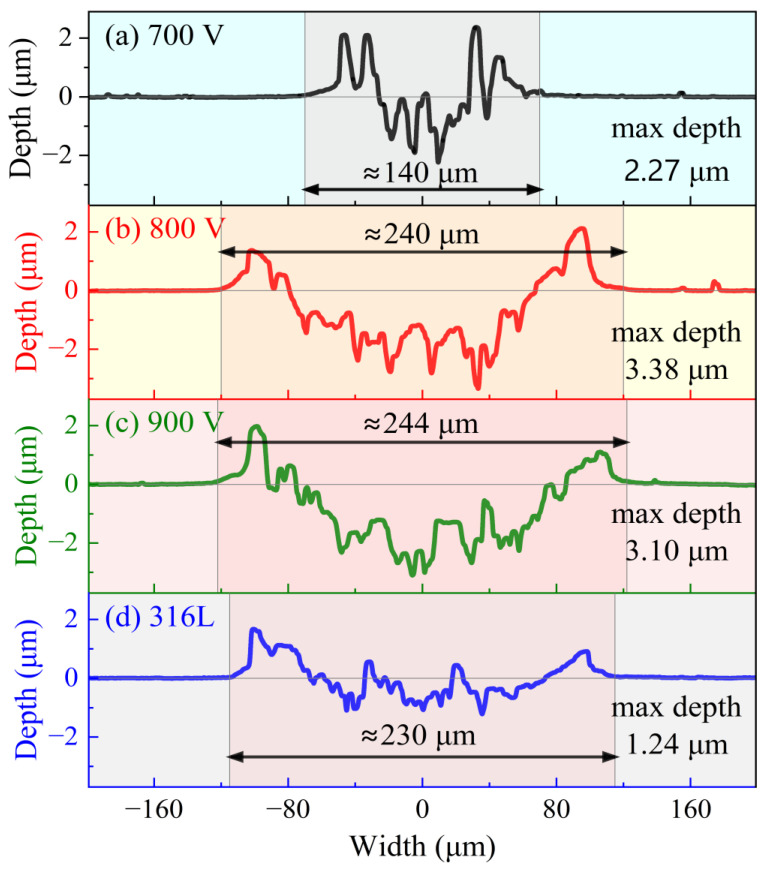
Cross-sectional profiles of wear tracks for (**a**) TiNbCr-700 V, (**b**) TiNbCr-800 V, (**c**) TiNbCr-900 V, and (**d**) 316L after wear tests under a load of 0.5 N, speed of 3.77 cm/s, and sliding distance of 40 m.

The wear test results presented in Figure 11 correlate well with the aforementioned mechanical properties and residual stress analyses. The sample deposited at 700 V exhibited superior wear resistance, characterized by the narrowest wear track width (~140 μm) and a moderate depth (2.27 μm). This performance is attributed to the highest residual compressive stress, which effectively improves the load-bearing capacity and restrains lateral plastic flow during sliding. The dense and defect-free microstructure also contributes to mild abrasive wear, leading to the narrowest wear track. Such a balance effectively inhibited crack initiation and propagation, maintaining the wear mechanism within a mild abrasive regime.

In contrast, samples deposited at 800 V and 900 V displayed significantly wider (~240 μm) and deeper (>3 μm) wear tracks. In contrast, samples deposited at 800 V and 900 V displayed significantly wider (~240 μm) and deeper (>3 μm) wear tracks. For the 800 V sample, although it has the highest hardness and optimal *H*/*E* ratio, the lower residual compressive stress weakens the surface support effect and leads to local brittle fracture during sliding. For the 900 V sample, the lowest hardness and residual stress result in poor load-bearing capacity. These factors cause the wear mechanism to shift to severe fatigue spalling.

In contrast, samples deposited at 800 V and 900 V displayed significantly wider (~240 μm) and deeper (>3 μm) wear tracks. For the 800 V sample, although it has the highest hardness and optimal *H*/*E* ratio, the lower residual compressive stress weakens the surface support effect and leads to local brittle fracture during sliding. For the 900 V sample, the lowest hardness and residual stress result in poor load-bearing capacity. These factors cause the wear mechanism to shift to severe fatigue spalling. These findings underscore the critical role of optimizing trigger voltage to balance residual stress, hardness, and load-bearing capacity for achieving improved tribological performance. The tribological behavior is jointly dominated by the synergistic effect of residual stress and hardness, rather than a single mechanical parameter.

### 3.5. Corrosion Resistance of Ti-Nb-Cr Thin Film

**Figure 12:**
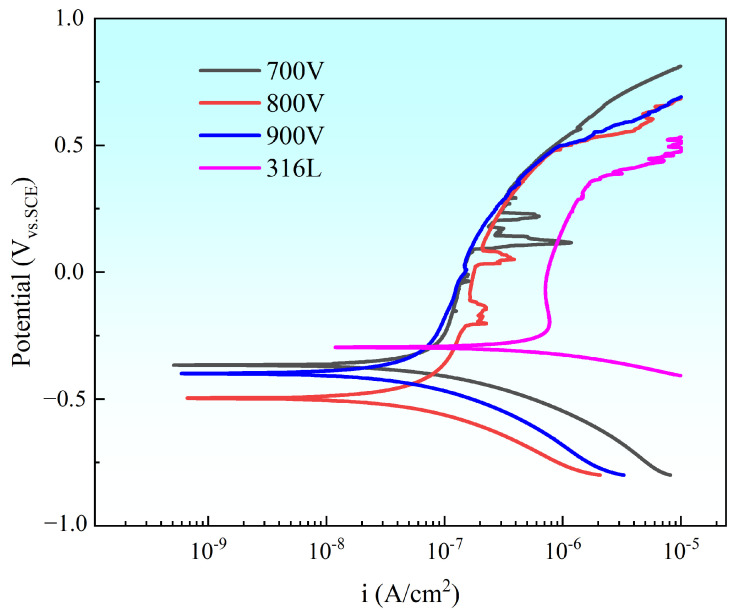
Polarization curves of Ti-Nb-Cr films deposited at different peak powers.

To evaluate the corrosion resistance, potentiodynamic polarization curves of Ti-Nb-Cr thin films prepared at different triggering voltages (700, 800, and 900 V) were measured in a NaCl (mass fraction 3.5%) solution, as shown in Figure 12. The bare 316L substrate was used as the control group. It can be seen that all samples exhibited an obvious passivation zone. Notably, compared to the 316L substrate, the Ti-Nb-Cr films demonstrated a significantly broader passivation range. This observation suggests that Ti-Nb-Cr films possess higher stability and superior protective properties.

To quantitatively evaluate the corrosion resistance, the corrosion potential (E_corr_) and corrosion current density (I_corr_) of the sample were derived from the polarization curves using the Tafel extrapolation method. The corresponding values are summarized in Table 2.

Compared to the bare 316L substrate, two primary conclusions can be drawn from the data in Table 2. Firstly, all Ti-Nb-Cr films exhibit more negative Ecorr, indicating a higher thermodynamic corrosion tendency. This observation suggests that the coated surfaces are more electrochemically active than the bare substrate. Secondly, the Icorr values of all Ti-Nb-Cr films are reduced by one order of magnitude, suggesting improved corrosion resistance. This can be primarily attributed to (1) the robust physical barrier effect of the dense and stable passive films that prevents corrosive electrolyte (e.g., Cl^−^) from penetrating through the coating to reach the underlying substrate and (2) the amorphous structure (as evidenced by the XRD results in Figure 4) that eliminates preferential corrosion paths such as grain boundaries.

Among the Ti-Nb-Cr films, Ecorr shows a nonmonotonic variation with trigger voltage, following the order 800 V (most negative) < 900 V (intermediate) < 700 V (most positive). The most negative E_corr_ of the 800 V sample may be attributed to its longitudinal rock-like columnar structure and noticeable cross-sectional undulation (Figure 6), features indicative of incomplete densification. As E_corr_ reflects thermodynamic tendency rather than the actual corrosion rate, the analysis below focuses on I_corr_, the key indicator of corrosion resistance.

I_corr_ decreases slightly from 2.95 × 10^−8^ A·cm^−2^ (700 V) to 2.67 × 10^−8^ A·cm^−2^ (800 V) and further to 1.91 × 10^−8^ A·cm^−2^ (900 V). These values are all within the same order of magnitude (10^−8^ A·cm^−2^), and the differences among them are relatively small, indicating that all coatings provide effective corrosion protection.

The 700 V sample, deposited at the lowest ion atomic arrival ratios, consists of fine, dispersed nanocrystalline grains (Figure 6). It exhibits the highest residual compressive stress (Figure 8) and lower hardness, toughness, and resistance to plastic deformation (Figure 9). The limited ion flux under this condition leads to insufficient atomic rearrangement and a less dense structure, providing preferential pathways for electrolyte penetration and thus yielding the weakest barrier effect, which accounts for the highest I_corr_.

In contrast, the 900 V sample, with the highest ion atomic arrival ratios, presents a denser columnar structure with significantly reduced undulation compared to the 800 V sample (Figure 6), exhibiting a more uniform and denser morphology. The high ion flux promotes atomic diffusion and reconstruction, leading to a dense and uniform columnar structure. Additionally, this sample has the largest film thickness (Figure 6). The enhanced density and increased thickness collectively provide the most effective barrier against electrolyte penetration, resulting in the lowest Icorr and optimal corrosion resistance. The 800 V sample, with intermediate ion atomic arrival ratios, exhibits a longitudinal rock-like columnar structure with more pronounced undulation (Figure 6) and optimal mechanical properties (Figure 9), which contribute to a barrier effect stronger than that of the 700 V sample but weaker than that of the 900 V sample, resulting in an intermediate I_corr_.

It is worth noting that even the Ti-Nb-Cr films deposited at 700 V and 800 V possess sufficiently dense microstructures to provide effective corrosion protection. The advantage of the 900 V sample lies in its more uniform bulk structure and greater thickness, which further enhances barrier performance.

## 4. Conclusions

In this study, Ti-Nb-Cr thin films were fabricated using high-power impulse magnetron sputtering (HiPIMS). The plasma characteristics, microstructure, mechanical properties, and corrosion resistance of the films were systematically investigated, leading to the following conclusions:

(1) As the peak power increased from 39.44 kW to 67.84 kW, ion atomic arrival ratios significantly improved. Specifically, the ion atomic arrival ratio of Ti increased from 20.9% to 47.57%, while those for Nb and Cr rose from 25.6% to 39.4% and from 5.8% to 10.6%, respectively.
(2) The deposited Ti-Nb-Cr films exhibited a predominantly amorphous structure.
(3) At higher power levels, the TiNbCr films exhibit reduced residual compressive stress, although this may be accompanied by lower hardness and wear resistance. The hardness, elastic modulus, corrosion performance, and wear characteristics do not follow a corresponding predictable trend, and no definitive correlation exists among these properties.

## Figures and Tables

**Table 1:** Deposition parameters of the TiNbCr films.

Sample	Peak Power /kW	Trigger Voltage /V	Peak Current /A	Average Power /W	Pulse Width /μs	Ar/ (sccm)	Pressure /Pa	Film Thickness /nm
TNC-700	51.04	700	88	3306	100	50	0.4	743 ± 64
TNC-800	39.44	800	68	2691				871 ± 60
TNC-900	67.84	900	106	4966				966 ± 55

**Table 2:** Corrosion potential and corrosion current density of Ti-Nb-Cr thin films.

Sample	Peak Power (kW)	E_corr_ (V_vs.SCE_)	I_corr_ (A·cm^−2^)
TiNbCr-700 V	51.04	−0.367	2.95 × 10^−8^
TiNbCr-800 V	39.44	−0.497	2.67 × 10^−8^
TiNbCr-900 V	67.84	−0.4	1.91 × 10^−8^
316L	-	−0.297	3.25 × 10^−7^

## Data Availability

The original contributions presented in this study are included in the article. Further inquiries can be directed to the corresponding author.

## Abbreviations

The following abbreviations are used in this manuscript:

HiPIMS	High-power impulse magnetron sputtering
OES	Optical emission spectroscopy
XRD	X-ray diffraction
SEM	Scanning electron microscopy
TEM	Transmission electron microscopy
